# Significant Differences in Bacterial and Potentially Pathogenic Communities Between Sympatric Hooded Crane and Greater White-Fronted Goose

**DOI:** 10.3389/fmicb.2019.00163

**Published:** 2019-02-05

**Authors:** Xingjia Xiang, Fengling Zhang, Rong Fu, Shaofei Yan, Lizhi Zhou

**Affiliations:** ^1^Anhui Province Key Laboratory of Wetland Ecological Protection and Restoration, School of Resources and Environmental Engineering, Anhui University, Hefei, China; ^2^Anhui Biodiversity Information Center, Hefei, China

**Keywords:** migratory bird, sequencing, wetland, intestinal bacteria, pathogen

## Abstract

The gut microbiota of vertebrates play a crucial role in shaping the health of their hosts. However, knowledge of the avian intestinal microbiota has arguably lagged behind that of many other vertebrates. Here, we examine the intestinal bacterial communities of the hooded crane and the greater white-fronted goose at the Shengjin Lake of China, using high-throughput sequencing (Illumina Mi-Seq), and infer the potential pathogens associated with each species. Intestinal bacterial alpha-diversity in the greater white-fronted goose was significantly higher than that in hooded crane. The intestinal bacterial community compositions were significantly different between the two hosts, suggesting that host interactions with specific communities might have profound implications. In addition, potential pathogens were detected in both guts of the two hosts, suggesting that these wild birds might be at risk of disease and probably spread infectious disease to other sympatric vertebrates. The gut of hooded crane carried more potential pathogens than that of the greater white-fronted goose. The potentially pathogenic community compositions were also significantly different between the two hosts, suggesting the divergence of potentially pathogenic communities between hooded crane, and greater white-fronted goose. Finally, bacterial and potentially pathogenic structures showed strong evidence of phylogenic clustering in both hosts, further demonstrating that each host was associated with preferential and defined bacterial and potentially pathogenic communities. Our results argue that more attention should be paid to investigate avian intestinal pathogens which might increase disease risks for conspecifics and other mixed species, and even poultry and human beings.

## Introduction

The gut microbiota of vertebrates is one of the most densely populated microbial assemblages (Whitman et al., 1998), and plays an essential role in the health of their hosts (Heijtza et al., 2011). The intestinal microbes contribute to many necessary functions for their hosts, including aiding in digestion (Turnbaugh et al., 2006; Stanley et al., 2012), vitamin synthesis and metabolism (Eberl and Boneca, 2010), protection against pathogens (Guarner and Malagelada, 2003; Koch and Schmid-Hempel, 2011), training of the immune system (Atarashi et al., 2011; Chung et al., 2012), organ development (Stappenbeck et al., 2002; Rahimi et al., 2009), and regulation of host physiology (Backhed et al., 2004; Meinl et al., 2009). The microbiota may even affect mate choice and induce hybrid inviability (Sharon et al., 2010; Brucker and Bordenstein, 2013). In vertebrates, the intestinal microbial assemblage patterns are shaped by a series of complex and dynamic interactions throughout life, including diet (De Filippo et al., 2010), lifestyle (Ley et al., 2008; Nicholson et al., 2012), and seasonal fluctuations (Hird et al., 2014). Intestinal microbial communities are largely shaped by their host species, and microbial communities tend to be more similar between more closely related hosts (Eckburg et al., 2005).

Birds represent an interesting study system for intestinal microbes because they have unique life history traits and developmental strategies that are different from other vertebrates (Kohl, 2012). However, research of the avian intestinal microbiota has arguably lagged behind that of many other vertebrates. Recent studies of avian intestinal microbiota are mainly focused on ornamental and economically important birds (e.g., kakapo, hoatzins, poultry, etc), most of which showed that gastrointestinal microbial communities bring benefits to their hosts (Jin et al., 1998; Angelakis and Raoult, 2010; Torok et al., 2011; Zhang et al., 2011; Cao et al., 2012; Stanley et al., 2012). However, there are also pathways through which the colonization of intestinal microbes might be of detriment, triggering a series of avian diseases (Ford and Coates, 1971; Potti et al., 2002; Cao et al., 2012; Singh et al., 2013). Migratory birds travel long distances and utilize diverse habitats, which potentially exposes them to a broad range of pathogens and could spread infectious disease to conspecifics and/or other bird species, or even poultry and human beings (Altizer et al., 2011). However, the assumption that migrating birds facilitate pathogenic propagation has not been definitely verified.

The hooded crane (*Grus monacha*) and greater white-fronted goose (*Anser albifrons*) are two large long-distance migratory colonial wading wild birds. The hooded crane is defined as a vulnerable species in the IUCN (International Union for Conservation of Nature and Natural Resources) Red List of Threatened Species and is a first-class national protected wild animal in China, breeding in south-central and south-eastern Siberia and Russia, and wintering in China, Japan, and South Korea (Zheng et al., 2015). The East Asia greater white-fronted goose breeds mainly on the Siberian arctic coast, and hibernates in China, India, and Japan. The greater white-fronted goose is one of the most abundant wintering bird in the Yangtze River floodplain. However, in recent decades, the wintering population of greater white-fronted geese has decreased markedly (Cao et al., 2008), with the population falling from around 140,000 in 1987 to about 18,000 in 2010 due to habitat loss and hydrological changes (Zhao et al., 2012). The wintering period of these two migratory birds is from October to April in the Yangtze River floodplain. Anthropic activities trigger rapid degradation of lake wetlands, leading to the significant reduction in food availability, which forces wintering birds to change their dietary structure and flock together for foraging (Barzen et al., 2009; Zhou et al., 2010; Yang et al., 2015).

Shengjin Lake, an internationally important wetland, is a river-connected shallow lake in the middle of the Yangtze River floodplain (Fang et al., 2006). Shengjin Lake is the most important wintering ground for hooded cranes and greater white-fronted geese, providing them with suitable feeding habitats during the wintering period (Chen et al., 2011). Previous studies have demonstrated that hooded cranes and greater white-fronted geese forage together in this area (Chen et al., 2011; Yang et al., 2015; Zheng et al., 2015), which offered the opportunity to compare intestinal bacterial and potentially pathogenic communities between these two hosts. A better understanding of intestinal microbes as well as pathogens of wild birds is important for clarifying avian ecology and disease propagation. In this study, high-throughput sequencing method (Illumina Mi-Seq) was used to analyze the intestinal bacterial communities of wintering hooded crane and greater white-fronted goose at the Shengjin Lake. In particular, we want to examine the bacterial communities and infer the potential pathogens in the guts of these two hosts.

## Materials and Methods

### Ethics Statement

Fecal samples of hooded crane and greater white-fronted goose were collected after foraging to avoid human disturbance. Non-invasive sample collection did not involve the hunting of experimental animals. Permission was obtained from the Shengjin Lake National Nature Reserve of Anhui Province.

### Site Selection and Sample Collection

The Shengjin Lake (30.15–30.30°N, 116.55–117.15°E) is a river-connected shallow lake, which flows into the Yangtze River (Supplementary Figure S1). The lake is an internationally important wetland, which serves as indispensable wintering and stopover habitat for migratory birds on the East Asia-Australasian flyway (Chen et al., 2011; Fox et al., 2011). The average annual temperature and precipitation are 16.14°C and 1600 mm, respectively (Fang et al., 2006).

Fecal samples from hooded crane and greater white-fronted goose were collected on the 10th of March, 2018 at the Shegan, Shengjin Lake (Supplementary Figure S1). Hooded crane mainly eats *Vallisneria natans* and *Potamogeton malaianus* (Zheng et al., 2015) while greater white-fronted goose feeds primarily on *Carex spp.* (Zhang and Lu, 1999; Cheng et al., 2009) in the early wintering period (i.e., from October to next January) at Shengjin Lake. However, the wading birds alter their dietary structure to exploit paddy fields as foraging habitat due to food shortage in the later wintering period (i.e., February to April; Zhou et al., 2010; Yang et al., 2015). There are lots of paddy fields around the Shegan region, so hooded cranes and greater white-fronted geese forage together here.

Before sampling, a telescope was used to search the flocks of hooded crane and greater white-fronted goose. The fresh fecal samples were collected immediately after foraging of wild birds. The interval distance for samples was more than 5 meters to avoid individual repetition. The fecal samples were kept in a cooler and transported refrigerated to the lab as quickly as possible. The outside of each sample was cut and discarded to avoid contamination; the rest was homogenized within each plastic valve bag and stored at -20°C for DNA extraction.

### Fecal DNA Extraction

DNA extractions were carried out on 200 mg of fecal samples using the Qiagen QIAamp^®^ DNA Stool Mini Kit following the DNA isolation protocol. The extracted DNA was dissolved in 60 μl of elution buffer, quantified by NanoDrop ND-1000 (Thermo Scientific, United States), and stored at -20°C.

### Bird Species Determination

Primer sets BIRDF1–BIRDR1 were used to amplify COL gene to confirm bird species (Hebert et al., 2004). PCR reaction was carried out in 50 μl reaction mixtures containing 100 ng of fecal DNA, each deoxynucleoside triphosphate at a concentration of 200 μM, forward or reverse primers at a concentration of 0.4 μM and 2 U of Taq DNA polymerase (TaKaRa, Japan). The cycling parameters were as follows: 95°C for 5 min, followed by 35 cycles of 95°C for 30 s, 55°C for 45 s, and 72°C for 90 s, with a final extension period at 72°C for 10 min. The PCR products were sequenced and then blasted (>97% sequence identity) in National Center for Biotechnology Information (NCBI). Only the fecal sample with sequence belonged to hooded crane or greater white-fronted goose was kept for high-throughput sequencing. A total of 30 fecal samples, 15 from hooded crane and 15 from greater white-fronted goose, were determined in this study.

### PCR and Amplicon Library Preparation

An aliquot (50 ng) of purified DNA from each sample was used as template for amplification. Primer sets F515/R907 equipped with sequencing adapters and unique identifier tags were used to amplify the V4-V5 hypervariable regions of the bacterial 16S rRNA genes fragments (Biddle et al., 2008) for the Illumina Mi-Seq platform (PE 300) at Majorbio (Shanghai, China). PCR reaction was carried out in 50 μl reaction mixtures containing each deoxynucleoside triphosphate at a concentration of 200 μM, forward or reverse primers at a concentration of 0.4 μM and 2 U of Taq DNA polymerase (TaKaRa, Japan). The following cycling parameters were used: 35 cycles of denaturation at 94°C for 45 s, annealing at 55°C for 45 s, and extension at 72°C for 45 s; with a final extension at 72°C for 10 min. To check for contamination, PCR negative controls were performed without added DNA template. Negative PCR controls did not contain detectable PCR product and were not processed for sequencing. Triplicate reaction mixtures per sample were pooled together and purified using an agarose gel DNA purification kit (TaKaRa). The PCR products were pooled in equimolar amounts (10 pg for each sample) before sequencing.

### Processing of Sequence Data

Bacterial raw data were processed by the Quantitative Insights Into Microbial Ecology (QIIME v.1.9; Caporaso et al., 2010). The poor-quality sequences (below an average quality score of 30 and the length <250 bp) were removed. High quality sequences were clustered into Operational Taxonomic Units (OTUs; 97% similarity; *de novo* approach) using UCLUST (Edgar, 2010). Chimera and singleton OTUs were deleted. The most abundant sequence within each OTU was selected as the representative sequence identified by the ribosomal database project Classifier (Wang et al., 2007), and aligned by PyNAST (Caporaso et al., 2010). To equally rarefy samples, randomly selected subsets of 5,600 sequences (lowest sequence read depth; repetition with 20 times) per sample were used to compare bacterial community compositions and diversity for all samples.

### Potentially Pathogenic Species Determination

All identified bacterial species were manually searched as key words in Web of Science. These bacterial species which have been demonstrated by references as pathogens in human and/or animals were set aside for further analysis. A total of 11 potentially pathogenic species have been detected in this study (Supplementary Table S1). The *Clostridium perfringens* might cause disease in humans, birds, pigs, dogs, goats, etc. (Craven et al., 2000; Songer, 2010; Mafruza et al., 2012; Kiu and Hall, 2018; Liu et al., 2018). The *Prevotella copri* and *Staphylococcus aureus* probably invade humans and mice (Scher et al., 2013; Tong et al., 2015). The *Helicobacter pylori*, *Elizabethkingia meningoseptica*, *Bacillus cereus*, and *Prevotella nigrescens* are mainly human pathogens (Kotiranta et al., 2000; Kusters et al., 2006; Stingu et al., 2013; Jean et al., 2014). Fish are the primary hosts for *Flavobacterium columnare* and *Piscirickettsia salmonis* (Durborrow et al., 1998; Smith et al., 1999). The *Plesiomonas shigelloides* might be a pathogen in humans and fish (Claesson et al., 1984; Hu et al., 2014; Behara et al., 2018). The *Mucispirillum schaedleri* might cause disease in mice (Loy et al., 2017).

### Statistical Analysis

Identification of intestinal bacterial taxa that differed significantly between host species was performed by linear discriminant analysis (LDA) effect size (LEfSe), which uses the non-parametric Kruskal-Wallis rank sum test with the default setting (an alpha value of 0.05 and an effect size threshold of 2) to identify biomarkers (Segata et al., 2011). The differences in bacterial and pathogenic community compositions between host species were analyzed by non-metric multidimensional scaling (NMDS) and analysis of similarity (ANOSIM; permutations = 999) using the vegan package (Version 2.0-2; Oksanen et al., 2011) in R v.2.8.1 (R Development Core Team, 2006). The nearest taxon index (NTI) was calculated to test phylogenetic structure using the picante package (Purcell et al., 2007) in R v.2.8.1 (R Development Core Team, 2006). More positive NTI values indicate phylogenetic clustering, while more negative NTI values indicate phylogenetic overdispersion (Webb, 2000). One-way ANOVA was used to analyze alpha-diversity and NTI values which followed normal distribution across samples (Kolmogorov-Smirnov test; Supplementary Table S2). The Mann-Whitney-Wilcoxon test was used to analyze the relative abundance of pathogenic species which followed non-normal distribution (Kolmogorov-Smirnov test; Supplementary Table S2).

### Data Availability

The raw data were submitted to the Sequence Read Archive (SRA) of NCBI under the accession number SRP159542.

## Results

### Intestinal Bacterial Alpha-Diversity

**FIGURE 1:**
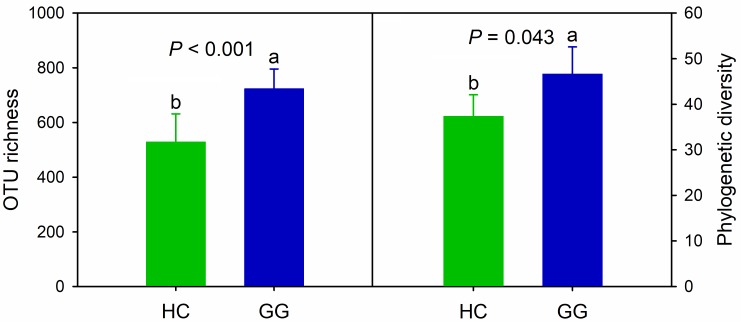
Intestinal bacterial alpha-diversity in hooded crane and greater white-fronted goose. Bars represent mean; error bars denote standard deviation; letters above bars represents significant differences from one-way ANOVA (*P* < 0.05). HC, hooded crane; GG, greater white-fronted goose; OTU, operational taxonomic unit; PD, phylogenetic diversity. The units of OTU richness and PD are number of OTUs and branch length of phylogenetic tree, respectively.

We obtained a total of 443,460 quality-filtered bacterial sequences across all samples for the primer pair F515/R907, ranging from 5622 to 25132 sequences per sample (Supplementary Table S3). A total of 5,325 bacterial OTUs was found, ranging from 265 to 885 across all samples (97% similarity), 32.3% of which (1722) were found in both species. The unique bacterial OTUs were 1545 (29.0%) and 2058 (38.6%) for the hooded crane and greater white-fronted goose, respectively. One-way ANOVA showed that bacterial alpha-diversity in the gut of the greater white-fronted goose was significantly higher than that of the hooded crane (Figure 1).

### Intestinal Bacterial Community Structure

**FIGURE 2:**
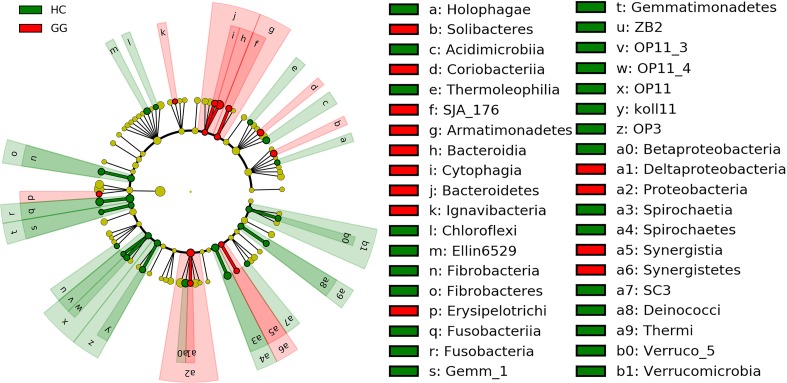
LEfSe analysis of intestinal bacterial biomarkers associated with host types. Identified phylotype biomarkers ranked by effect size and the alpha value was <0.05. Each filled circle represents one biomarker. Cladogram representing the taxonomic hierarchical structure of the phylotype biomarkers identified between two host types; green, phylotypes overrepresented in gut of hooded crane; red, phylotypes statistically overrepresented in gut of greater white-fronted goose; yellow, phylotypes for which relative abundance is not significantly different between the two host types. HC, hooded crane; GG, greater white-fronted goose.

The dominant intestinal bacterial phyla were Firmicutes (79.60%), Proteobacteria (11.83%), Bacteroidetes (4.71%), and Actinobacteria (1.21%). The dominant intestinal bacterial classes were *Clostridia* (77.33%), *Alphaproteobacteria* (8.60%), *Bacteroidia* (4.47%), *Bacilli* (2.20%), and *Gammaproteobacteria* (1.72%). LEfSe analysis identified specific intestinal bacterial taxa that were differentially abundant across the two hosts. The results showed that bacteria in eight phyla (i.e., Fibrobacteres, Fusobacteria, Gemmatimonadetes, OP11, OP3, Spirochaetes, Thermi and Verrucomicrobia), and 16 classes (i.e., *Holophagae*, *Acidimicrobiia*, *Thermoleophilia*, *Chloroflexi*, *Ellin6529*, *Fibrobacteria*, *Fusobacteriia*, *Gemm_1*, *ZB2*, *OP11_3*, *OP11_4*, *Koll11*, *Spirochaetia*, *SC3*, *Deinococci* and *Verruco_5*) were significantly more abundant in the gut of the hooded crane (Figure 2 and Supplementary Figure S2). Soil bacteria from four phyla (i.e., Armatimonadetes, Bacteroidetes, Proteobacteria, and Synergistetes) and nine classes (i.e., *Soilbacteres*, *Coriobacteriia*, *SJA_176*, *Bacteroidia*, *Cytophagia*, *Ignavibacteria*, *Erysipelotrichi*, *Synergistia*, and *Deltaproteobacteria*) were significantly more abundant in the gut of the greater white-fronted goose (Figure 2 and Supplementary Figure S2).

**FIGURE 3:**
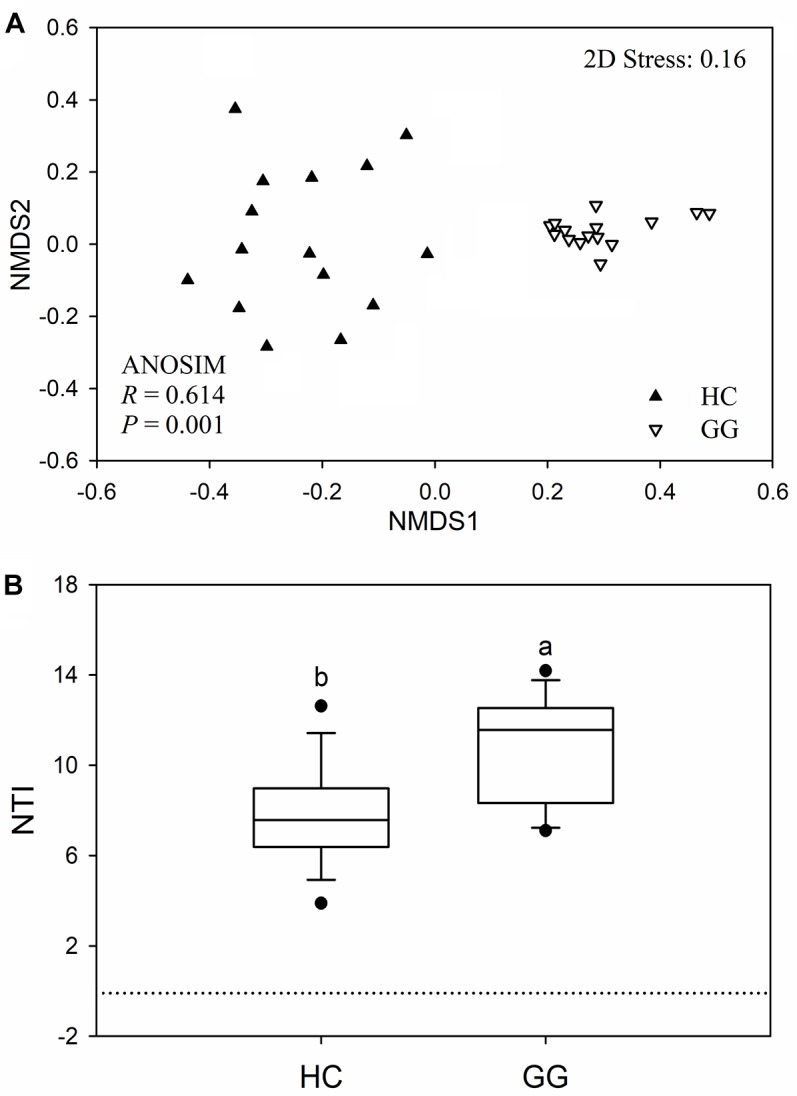
The intestinal bacterial community compositions **(A)** and the values of nearest taxon index (NTI; **B**) in guts of two hosts. HC, hooded crane; GG, greater white-fronted goose; ANOSIM, analysis of similarity.

The bacterial community compositions were significantly different between the guts of the hooded crane and the greater white-fronted goose (ANOSIM: *P* = 0.001; Figure 3A). The bacterial Bray-Curtis dissimilarity within host species was bigger in the gut of the hooded crane than in the greater white-fronted goose (Supplementary Figure S3). The NTI was calculated to test the bacterial phylogenetic structure in the two species. The NTI values were positive for all samples tested, which showed that bacterial communities were phylogenetically clustered in both hooded crane and greater white-fronted goose (Figure 3B). In addition, NTI was lower in the gut of the hooded crane than in the greater white-fronted goose, which indicated that phylogenetic clustering was weaker in the gut of the hooded crane than in the greater white-fronted goose (Figure 3B).

### Intestinal Potential Pathogen

**FIGURE 4:**
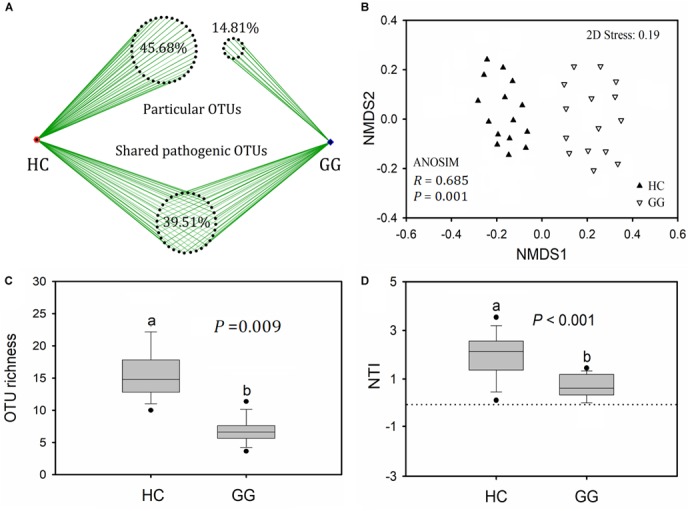
The intestinal pathogenic bacterial characteristics in guts of two hosts: pathogenic OTU overlapping **(A)**, operational taxonomic unit (OTU) richness **(B)**, community composition **(C),** and nearest taxon index (NTI, **D**). HC, hooded crane; GG, greater white-fronted goose; ANOSIM, analysis of similarity.

A total of 6168 (1.39% relative to all bacterial reads) potentially pathogenic sequences were found across all samples, ranging from 7 to 910 sequences per sample (Supplementary Table S3). Potentially pathogenic sequences were significantly higher in the gut of the hooded crane than in the greater white-fronted goose. These sequences grouped into 81 potentially pathogenic OTUs, 39.51% of which (32) were found in both host species. The gut of the hooded crane (37) had more unique pathogenic OTUs than the greater white-fronted goose (12, Figure 4A). One-way ANOVA showed that potentially pathogenic OTU richness was significantly higher in the gut of the hooded crane than in the greater white-fronted goose (Figure 4B).

A total of 11 potentially pathogenic species was detected in the guts of the hooded crane and the greater white-fronted goose. The primary dominant pathogenic species was *C. perfringens* which might be detrimental for birds (Supplementary Table S1). The other potential pathogens might cause diseases in human and/or specific animal species (Supplementary Table S1). In addition, the hooded crane gut carried more relative abundance of *C. perfringens* and *M. schaedleri* than in the greater white-fronted goose. The relative abundance of *P. copri* and *P. shigelloides* were significantly higher in the gut of the greater white-fronted goose than in the hooded crane (Supplementary Table S4). The potentially pathogenic community compositions were significantly different between hooded crane and greater white-fronted goose (ANOSIM: *P* = 0.001; Figure 4C). The potentially pathogenic NTI values were positive for all the samples, indicating that potentially pathogenic communities were also phylogenetically clustered in both the guts of hooded crane and greater white-fronted goose (Figure 4D). In addition, potentially pathogenic phylogenetic clustering was stronger in the gut of the hooded crane than in the greater white-fronted goose (Figure 4D).

## Discussion

In this study, we found significant differences in the intestinal bacterial community composition and diversity between hooded crane and greater white-fronted goose, demonstrating that bacterial taxa showed strong host-preference, suggesting that hosts were the crucial factor in shaping the intestinal bacterial structure (Eckburg et al., 2005). Previous study has shown that heritable taxa might be a reason to cause the shift in bacterial communities between different hosts (Goodrich et al., 2014). In this study, we found strong evidence for phylogenetic clustering of bacterial communities in both guts of the hooded crane and the greater white-fronted goose (Figure 3B), suggesting that different hosts were associated with specific and defined intestinal bacterial communities, which might be included by hosts mediated metabolic pathways and/or dietary selection (Nicholson et al., 2012; Goodrich et al., 2014).

The primary dominant intestinal bacterial phylum was Firmicutes (79.60%) in both the hooded crane and the greater white-fronted goose, which was consistent with prior studies in other birds, such as the chicken (Lan et al., 2002), seabirds penguin (Dewar et al., 2013, 2014) and turkey (Wilkinson et al., 2017). Intestinal Firmicutes contributes to the decomposition of complex carbohydrates, polysaccharides and fatty acids (Flint et al., 2008), which improves hosts’ ability to extract nutrients from food (Tap et al., 2009). A high proportion of Proteobacteria (11.83%), which played an important role in energy accumulation, was found in both guts of hooded crane and white-fronted goose (Chevalier et al., 2015), indicating that these wintering birds might consume lots of energy to deal with frost during wintering periods.

In this study, only 39.51% of total potentially pathogenic OTUs was found in both guts of the hooded crane and the greater white-fronted goose (Figure 4A). In addition, these two hosts carried significantly different potentially pathogenic compositions and diversity (Figure 4), suggesting the divergence of potentially pathogenic communities between hooded crane and greater white-fronted goose. We also found an interesting result where the hooded crane carried less bacterial diversity and more potentially pathogenic diversity relative to the greater white-fronted goose. Previous studies demonstrated that healthy mice contained more bacterial diversity while disease reduced a host’s intestinal bacterial diversity (Manichanh et al., 2006; de Vos and de Vos, 2012; Jeffery et al., 2012; Guan et al., 2016), indicating that there might be a negative relationship between intestinal bacterial and pathogenic diversity. Healthy hosts contain various bacterial groups while disease might break the balance of these groups to decrease bacterial diversity (Mangin et al., 2004).

The primary dominant potential pathogen was *C. perfringens* which might be detrimental for birds (Craven et al., 2000; Ryan and Ray, 2004; Songer, 2010; Mafruza et al., 2012; Liu et al., 2018), suggesting that these wild birds might be at risk of disease. Hooded crane harbored much more abundance of *C. perfringens* relative to greater white-fronted goose (Supplementary Table S4), indicating that wintering hooded crane might be suffering more severe pathogenic invasion. Hooded cranes are a vulnerable species in the IUCN Red List of Threatened Species, so much more attention should be paid to protect hooded cranes. The *C. perfringens* triggers tissue necrosis, bacteremia, emphysematous cholecystitis and gas gangrene, not only to infect avian species, but also human beings (Ryan and Ray, 2004; Songer, 2010). Particularly, three potential pathogens in the feces of the two hosts might cause severe diseases in fish (Supplementary Table S1). The Shengjin Lake is an important fish farming base and these pathogens in avian feces could easily enter into the lake. There were also several potential pathogens which might cause diseases in human and/or specific animal species (Supplementary Table S1). Because of the migration of birds, they might widely propagate their intestinal pathogens and increase the risk of disease in other animals, even human beings (Caron et al., 2010; Mora et al., 2013; Huang et al., 2014; Ekong et al., 2018).

In conclusion, intestinal bacterial as well as potentially pathogenic communities were significantly different between the hooded crane and the greater white-fronted goose. This work helps to build a more complete picture of intestinal microbial communities, as well as potentially pathogenic communities in migratory birds. However, there were certain limitations in this research. Only two bird species with 15 replicates were chosen for analysis. In addition, the intestinal bacterial communities of wild birds were studied within one wintering region rather than across multiple regions. Lastly, we did not show pathogenic interaction among wild birds along the wintering timescale to distinguish the degree of cross infection. These limitations should be clarified in future studies.

## Author Contributions

XX and LZ designed the experiments. XX, FZ, and RF completed the field sampling. FZ and XX performed the data analysis and prepared the figures. XX wrote the manuscript. SY and LZ contributed to the revision of manuscript.

## Conflict of Interest Statement

The authors declare that the research was conducted in the absence of any commercial or financial relationships that could be construed as a potential conflict of interest.

## References

[B1] Altizer S. Bartel R. Han B. A. (2011). Animal migration and infectious disease risk. *Science* 331 296–302. 10.1126/science.1194694 21252339

[B2] Angelakis E. Raoult D. (2010). The increase of *Lactobacillus* species in the gut flora of newborn broiler chicks and ducks is associated with weight gain. *PLoS One* 5:e10463. 10.1371/journal.pone.0010463 20454557 PMC2864268

[B3] Atarashi K. Tanoue T. Shima T. Imaoka A. Kuwahara T. Momose Y. (2011). Induction of colonic regulatory T cells by indigenous *Clostridium* Species. *Science* 331 337–341. 10.1126/science.1198469 21205640 PMC3969237

[B4] Backhed F. Ding H. Wang T. Hooper L. V. Koh G. Y. Nagy A. (2004). The gut microbiota as an environmental factor that regulates fat storage. *Proc. Natl. Acad. Sci. U.S.A.* 101 15718–15723. 10.1073/pnas.0407076101 15505215 PMC524219

[B5] Barzen J. A. Engels M. Burnham J. Harris J. Wu G. (2009). Potential impacts of a water control structure on the abundance and distribution of wintering waterbirds at poyang lake. *J. Jpn. Stat. Soc.* 38 293–309.

[B6] Behara B. K. Bera A. K. Paria P. Das A. Parida P. K. Susman Kumari (2018). Identification and pathogenicity of *Plesiomonas shigelloides* in silver carp. *Aquaculture* 493 314–318. 10.1016/j.aquaculture.2018.04.063 24818472

[B7] Biddle J. F. Fitz-Gibbon S. Schuster S. C. Brenchley J. E. House C. H. (2008). Metagenomic signatures of the peru margin subsea floor biosphere show a genetically distinct environment. *Proc. Natl. Acad. Sci. U.S.A.* 105 10583–10588. 10.1073/pnas.0709942105 18650394 PMC2492506

[B8] Brucker R. M. Bordenstein S. R. (2013). The hologenomic basis of speciation: gut bacteria cause hybrid lethality in the genus *Nasonia*. *Science* 341 667–669. 10.1126/science.1240659 23868918

[B9] Cao G. T. Xiao Y. P. Yang C. M. Chen A. G. Liu T. T. Zhou L. (2012). Effects of *Clostridium butyricum* on growth performance, nitrogen metabolism, intestinal morphology and cecal microflora in broiler chickens. *J. Anim. Vet. Adv.* 11 2665–2671. 10.3923/javaa.2012.2665.2671

[B10] Cao L. Barter M. Lei G. (2008). New anatidae population estimates for eastern China: implications for current flyway estimates. *Biol. Conserv.* 141 2301–2309. 10.1016/j.biocon.2008.06.022

[B11] Caporaso J. G. Kuczynski J. Stombaugh J. Bittinger K. Bushman F. D. Costello E. K. (2010). QIIME allows analysis of high-throughput community sequencing data. *Nat. Methods* 7 335–336. 10.1038/nmeth.f.303 20383131 PMC3156573

[B12] Caron A. Garine-Wichatitsky M. D. Gaidet N. Chiweshe N. Cumming G. S. (2010). Estimating dynamic risk factors for pathogen transmission using community-level bird census data at the wildlife/domestic interface. *Ecol. Soc.* 15 299–305. 10.5751/ES-03547-150325

[B13] Chen J. Y. Zhou L. Z. Zhou B. Xu R. X. Zhu W. Z. Xu W. B. (2011). Seasonal dynamics of wintering waterbirds in two shallow lakes along yangtze river in anhui province. *Zool. Res.* 32 540–548. 10.3724/SP.J.1141.2011.05540 22006808

[B14] Cheng Y. Q. Cao L. Barter M. Xu W. B. Zhang Y. Zhao M. J. (2009). *Wintering waterbird survey at the Anhui Shengjin Lake National Nature Reserve, China 2008/9.* Hefei: University of Science and Technology of China Press.

[B15] Chevalier C. Stojanović O. Colin D. J. Suarez-Zamorano N. Tarallo V. Veyrat-Durebex C. (2015). Gut microbiota orchestrates energy homeostasis during cold. *Cell* 163 1360–1374. 10.1016/j.cell.2015.11.004 26638070

[B16] Chung H. C. Pamp S. J. Hill J. A. Surana N. K. Edelman S. M. Troy E. B. (2012). Gut Immune maturation depends on colonization with a host-specific microbiota. *Cell* 149 1578–1593. 10.1016/j.cell.2012.04.037 22726443 PMC3442780

[B17] Claesson B. E. Holmlund D. E. Lindhagen C. A. Mätzsch T. W. (1984). *Plesiomonas shigelloides* in acute cholecystitis: a case report. *J. Clin. Microbiol.* 20 985–987. 6511879 10.1128/jcm.20.5.985-987.1984 PMC271489

[B18] Craven S. E. Stern N. J. Line E. Bailey J. S. Cox N. A. Fedorka-Cray P. (2000). Determination of the incidence of *Salmonella spp., Campylobacter jejuni*, and *Clostridium perfringens* in wild birds near broiler chicken houses by sampling intestinal droppings. *Avian Dis.* 44 715–720. 11007026

[B19] De Filippo C. Cavalieri D. Di Paola M. Ramazzotti M. Poullet J. B. Massart S. (2010). Impact of diet in shaping gut microbiota revealed by a comparative study in children from Europe and rural Africa. *Proc. Natl. Acad. Sci. U.S.A.* 107 14691–14696. 10.1073/pnas.1005963107 20679230 PMC2930426

[B20] de Vos W. M. de Vos E. A. J. (2012). Role of the intestinal microbiome in health and disease: from correlation to causation. *Nutr. Rev.* 70 S45–S56. 10.1111/j.1753-4887.2012.00505.x 22861807

[B21] Dewar M. L. Arnould J. P. Y. Dann P. Trathan P. Groscolas G. Smith S. (2013). Interspecific variations in the gastrointestinal microbiota in penguins. *Microbiologyopen* 2 195–204. 10.1002/mbo3.66 23349094 PMC3584224

[B22] Dewar M. L. Arnould J. P. Y. Krause L. Dann P. Smith S. C. (2014). Interspecific variations in the faecal microbiota of Procellariiform seabirds. *FEMS Microbiol. Ecol.* 89 47–55. 10.1111/1574-6941.12332 24684257

[B23] Durborrow R. M. Thune R. L. Hawke J. P. Camus A. C. (1998). *Columnaris Disease - A Bacterial Infection Caused by Flavobacterium columnare.* Washington, DC: SRAC Publication.

[B24] Eberl G. Boneca I. G. (2010). Bacteria and MAMP-induced morphogenesis of the immune system. *Curr. Opin. Immunol.* 22 448–454. 10.1016/j.coi.2010.06.002 20580214

[B25] Eckburg P. Bik E. M. Bernstein C. N. Purdom E. Dethlefsen L. Sargent M. (2005). Diversity of the human intestinal microbial flora. *Science* 308 1635–1638. 10.1126/science.1110591 15831718 PMC1395357

[B26] Edgar R. C. (2010). Search and clustering orders of magnitude faster than BLAST. *Bioinformatics* 26 2460–2461. 10.1093/bioinformatics/btq461 20709691

[B27] Ekong P. S. Fountain-Jones N. M. Alkhamis M. A. (2018). Spatiotemporal evolutionary epidemiology of H5N1 highly pathogenic avian influenza in West Africa and Nigeria, 2006-2015. *Transbound. Emerg. Dis.* 65 70–82. 10.1111/tbed.12680 28710829

[B28] Fang J. Wang Z. Zhao S. Li Y. Tang Z. Yu D. (2006). Biodiversity changes in the lakes of the central yangtze. *Front. Ecol. Environ.* 4:369–377.

[B29] Flint H. J. Bayer E. A. Rincon M. T. Lamed R. White B. A. (2008). Polysaccharide utilization by gut bacteria: potential for new insights from genomic analysis. *Nat. Rev. Microbiol.* 6 121–131. 10.1038/nrmicro1817 18180751

[B30] Ford D. J. Coates M. E. (1971). Absorption of glucose and vitamins of the B complex by germ-free and conventional chicks. *Proc. Nutr. Soc.* 30 10A–11A. 5090457

[B31] Fox A. D. Cao L. Zhang Y. Barter M. Zhao M. J. Meng F. J. (2011). Declines in the tuber-feeding waterbird guild at shengjin lake national nature reserve, China - a barometer of submerged macrophyte collapse. *Aquat. Conserv.* 21 82–91. 10.1007/s13280-018-1076-1 29987519 PMC6374229

[B32] Goodrich J. K. Waters J. L. Poole A. C. Sutter J. L. Koren O. Blekhman R. (2014). Human genetics shape the gut microbiome. *Cell* 159 789–799. 10.1016/j.cell.2014.09.053 25417156 PMC4255478

[B33] Guan G. Wang H. Chen S. Liu G. Xiong X. Tan B. (2016). Dietary chitosan supplementation increases microbial diversity and attenuates the severity of *Citrobacter rodentium* infection in mice. *Mediators Inflamm.* 2016:9236196. 10.1155/2016/9236196 27761062 PMC5059534

[B34] Guarner F. Malagelada J. R. (2003). Gut flora in health and disease. *Lancet* 361 512–519. 10.1016/S0140-6736(03)12489-0 12583961

[B35] Hebert P. D. N. Stoeckle M. Y. Zemlak T. S. Francis C. M. (2004). Identification of birds through DNA barcodes. *PLoS Biol.* 2:e312. 10.1371/journal.pbio.0020312 15455034 PMC518999

[B36] Heijtza R. D. Wang S. G. Anuar F. Qian Y. Bjorkholm B. Samuelsson A. (2011). Normal gut microbiota modulates brain development and behavior. *Proc. Natl. Acad. Sci. U.S.A.* 108 3047–3052. 10.1073/pnas.1010529108 21282636 PMC3041077

[B37] Hird S. M. Carstens B. C. Cardiff S. W. Dittmann D. L. Brumfield R. T. (2014). Sampling locality is more detectable than taxonomy or ecology in the gut microbiota of the brood-parasitic brown-headed cowbird (*Molothrus ater*). *PeerJ* 2:e321. 10.7717/peerj.321 24711971 PMC3970801

[B38] Hu Q. Lin Q. Shi C. Fu X. Li N. Liu L. (2014). Isolation and identification of a pathogenic *Plesiomonas shigelloides* from diseased grass carp. *Acta Microbiol. Sin.* 54 229–235. 24818472

[B39] Huang W. Zhou L. Z. Zhao N. N. (2014). Temporal-spatial patterns of intestinal parasites of the *Hooded crane* (*Grus monacha*) wintering in lakes of the middle and lower yangtze river floodplain. *Avian Res.* 5:6 10.1186/s40657-014-0006-6

[B40] Jean S. S. Lee W. S. Chen F. L. Ou T. Y. Hsueh P. R. (2014). *Elizabethkingia meningoseptica*: an important emerging pathogen causing healthcare-associated infections. *J. Hosp. Infect.* 86 244–249. 10.1016/j.jhin.2014.01.009 24680187

[B41] Jeffery I. B. O’Toole P. W. Öhman L. Claesson M. J. Deane J. Quigley E. M. (2012). An irritable bowel syndrome subtype defined by species-specific alterations in faecal microbiota. *Gut* 61 997–1006. 10.1136/gutjnl-2011-301501 22180058

[B42] Jin L. Z. Ho Y. W. Abdullah N. Ali M. A. Jalaludin S. (1998). Effects of adherent Lactobacillus cultures on growth, weight of organs and intestinal microflora and volatile fatty acids in broilers. *Anim. Feed Sci. Technol.* 70 197–209. 10.1016/S0377-8401(97)00080-1

[B43] Kiu R. Hall L. J. (2018). An update on the human and animal enteric pathogen *Clostridium perfringens*. *Emerg. Microbes Infect.* 7:141. 10.1038/s41426-018-0144-8 30082713 PMC6079034

[B44] Koch H. Schmid-Hempel P. (2011). Socially transmitted gut microbiota protect bumble bees against an intestinal parasite. *Proc. Natl. Acad. Sci. U.S.A.* 108 19288–19292. 10.1073/pnas.1110474108 22084077 PMC3228419

[B45] Kohl K. D. (2012). Diversity and function of the avian gut microbiota. *J. Comp. Physiol. B* 182 591–602. 10.1007/s00360-012-0645-z 22246239

[B46] Kotiranta A. Lounatmaa K. Haapasalo M. (2000). Epidemiology and pathogenesis of *Bacillus cereus* infections. *Microbes Infect.* 2 189–198. 10.1016/S1286-4579(00)00269-0 10742691

[B47] Kusters J. G. van Vliet A. H. M. Kuipers E. J. (2006). Pathogenesis of *Helicobacter pylori* Infection. *Clin. Microbiol. Rev.* 19 449–490. 10.1128/CMR.00054-05 16847081 PMC1539101

[B48] Lan P. T. N. Hayashi H. Sakamoto M. Benno Y. (2002). Phylogenetic analysis of cecal microbiota in chicken by the use of 16S rDNA clone libraries. *Microbiol. Immunol.* 46 371–382. 10.1111/j.1348-0421.2002.tb02709.x 12153114

[B49] Ley R. E. Hamady M. Lozupone C. Turnbaugh P. J. Ramey R. R. Bircher J. S. (2008). Evolution of mammals and their gut microbes. *Science* 320 1647–1651. 10.1126/science.1155725 18497261 PMC2649005

[B50] Liu N. Lin L. Wang J. Q. Zhang F. K. Wang J. P. (2018). Dietary cysteamine hydrochloride protects against oxidation, inflammation, and mucosal barrier disruption of broiler chickens challenged with *Clostridium perfringens*. *J. Anim. Sci.* 96 4339–4347. 10.1093/jas/sky292 30169609 PMC6162622

[B51] Loy A. Pfann C. Steinberger M. Hanson B. Herp S. Brugiroux S. (2017). Lifestyle and horizontal gene transfer-mediated evolution of *Mucispirillum schaedleri*, a core member of the murine gut microbiota. *mSystems* 2 e171–16. 10.1128/mSystems.00171-16 28168224 PMC5285517

[B52] Mafruza S. R. Sharma R. K. Borah P. Chakraborty A. Mandakini R. K. D. Longjam N. (2012). Characterization of *Clostridium perfringens* isolated from mammals and birds from Guwahati city. India. *J. Venom. Anim. Toxins* 18 83–87.

[B53] Mangin I. Bonnet R. Seksik P. Rigottier-Gois L. Sutren M. Bouhnik Y. (2004). Molecular inventory of faecal microflora in patients with crohn’s disease. *FEMS Microbiol. Ecol.* 50 25–36. 10.1016/j.femsec.2004.05.005 19712374

[B54] Manichanh C. Rigottier-Gois L. Bonnaud E. Gloux K. Pelletier E. Frangeul L. (2006). Reduced diversity of faecal microbiota in Crohn’s disease revealed by a metagenomic approach. *Gut* 55 205–211. 10.1136/gut.2005.073817 16188921 PMC1856500

[B55] Meinl W. Sczesny S. Brigelius-Flohe R. Blaut M. Glatt H. (2009). Impact of gut microbiota on intestinal and hepatic levels of phase 2 xenobiotic-metabolizing enzymes in the rat. *Drug Metab. Dispos.* 37 1179–1186. 10.1124/dmd.108.025916 19282396

[B56] Mora A. Viso S. López C. Alonso M. P. García-Garrote F. Dabhi G. (2013). Poultry as reservoir for extraintestinal pathogenic *Escherichia coli* O45:K1:H7-B2-ST95 in humans. *Vet. Microbiol.* 262 3–7. 10.1016/j.vetmic.2013.08.007 24008093

[B57] Nicholson J. K. Holmes E. Kinross J. Burcelin R. Gibson G. Jia W. (2012). Host-gut microbiota metabolic interactions. *Science* 336 1262–1267. 10.1126/science.1223813 22674330

[B58] Oksanen J. Blanchet G. Friendly M. Kindt R. Legendre P. McGlinn D. (2011). *Vegan: Community Ecology Package. Version 2.0–2.*

[B59] Potti J. Moreno J. Yorio P. Briones V. García-Borboroglu P. Villar S. (2002). Bacteria divert resources from growth for magellanic penguin chicks. *Ecol. Lett.* 5 709–714. 10.1046/j.1461-0248.2002.00375.x

[B60] Purcell D. Sompong U. Yim L. C. Barraclough T. G. Peerapornpisal Y. Pointing S. B. (2007). The effects of temperature, pH and sulphide on the community structure of hyperthermophilic streamers in hot springs of northern Thailand. *FEMS Microbiol. Ecol.* 60 456–466. 10.1111/j.1574-6941.2007.00302.x 17386034

[B61] Rahimi S. Grimes J. L. Fletcher O. Oviedo E. Sheldon B. W. (2009). Effect of a direct-fed microbial (Primalac) on structure and ultrastructure of small intestine in turkey poults. *Poult. Sci.* 88 491–503. 10.3382/ps.2008-00272 19211517

[B62] R Development Core Team (2006). *R: A language and Environment for Statistical Computing.* Vienna: Foundation for Statistical Computing.

[B63] Ryan K. J. Ray C. G. (2004). *Sherris Medical Microbiology: an Introduction to Infectious Diseases.* New York, NY: McGraw-Hill.

[B64] Scher J. U. Sczesnak A. Longman R. S. Segata N. Ubeda C. Bielski C. (2013). Expansion of intestinal *Prevotella copri* correlates with enhanced susceptibility to arthritis. *eLife* 2:e01202. 10.7554/eLife.01202 24192039 PMC3816614

[B65] Segata N. Izard J. Walron L. Gevers D. Miropolsky L. Garrett W. (2011). Metagenomic Biomarker Discovery and Explanation. *Genome Biol.* 12:R60. 10.1186/gb-2011-12-6-r60 21702898 PMC3218848

[B66] Sharon G. Segal D. Ringo J. M. Hefetz A. Zilber-Rosenberg I. Rosenberg E. (2010). Commensal bacteria play a role in mating preference of *Drosophila melanogaster*. *Proc. Natl. Acad. Sci. U.S.A.* 107 20051–20056. 10.1073/pnas.1009906107 21041648 PMC2993361

[B67] Singh P. Karmi A. Devendra K. Waldroup P. W. Cho K. K. Kwon Y. M. (2013). Influence of penicillin on microbial diversity of the cecal microbiota in broiler chickens. *Poult. Sci.* 92 272–276. 10.3382/ps.2012-02603 23243258

[B68] Smith P. A. Pizarro P. Ojeda P. Contreras J. Oyanedel S. Larenas J. (1999). Routes of entry of *Piscirickettsia salmonis* in rainbow trout *Oncorhynchus mykiss*. *Dis. Aquat. Organ.* 37 165–172. 10.3354/dao037165 10546046

[B69] Songer J. G. (2010). *Clostridia* as agents of zoonotic disease. *Vet. Microbiol.* 140 399–404. 10.1016/j.vetmic.2009.07.003 19682805

[B70] Stanley D. Denman S. E. Hughes R. J. Geier M. S. Crowley T. M. Chen H. (2012). Intestinal microbiota associated with differential feed conversion efficiency in chickens. *Appl. Microbiol. Biotechnol.* 96 1361–1369. 10.1007/s00253-011-3847-5 22249719

[B71] Stappenbeck T. S. Hooper L. V. Gordon J. I. (2002). Developmental regulation of intestinal angiogenesis by indigenous microbes via Paneth cells. *Proc. Natl. Acad. Sci. U.S.A.* 99 15451–15455. 10.1073/pnas.202604299 12432102 PMC137737

[B72] Stingu C. S. Schaumann R. Jentsch H. Eschrich K. Brosteanu O. Rodloff A. C. (2013). Association of periodontitis with increased colonization by *Prevotella nigrescens*. *J. Investig. Clin. Dent.* 4 20–25. 10.1111/j.2041-1626.2012.00129.x 22767485

[B73] Tap J. Mondot S. Levenez F. Pelletier E. Caron C. Furet J. P. (2009). Towards the human intestinal microbiota phylogenetic core. *Environ. Microbiol.* 11 2574–2584. 10.1111/j.1462-2920.2009.01982.x 19601958

[B74] Tong S. Y. Davis J. S. Eichenberger E. Holland T. L. Fowler V. G. (2015). *Staphylococcus aureus* infections: epidemiology, pathophysiology, clinical manifestations, and management. *Clin. Microbiol. Rev.* 28 603–610. 10.1128/CMR.00134-14 26016486 PMC4451395

[B75] Torok V. A. Hughes R. J. Mikkelsen L. L. Perez-Maldonado R. Balding K. Macalpine R. (2011). Identification and characterization of potential performance-related gut microbiotas in broiler chickens across various feeding trials. *Appl. Environ. Microbiol.* 77 5868–5878. 10.1128/AEM.00165-11 21742925 PMC3165380

[B76] Turnbaugh P. J. Ley R. E. Mahowald M. A. Magrini V. Mardis E. R. Gordon J. I. (2006). An obesity-associated gut microbiome with increased capacity for energy harvest. *Nature* 444 1027–1031. 10.1038/nature05414 17183312

[B77] Wang Q. Garrity G. M. Tiedje J. M. Cole J. R. (2007). Naive Bayesian classifier for rapid assignment of rRNA sequences into the new bacterial taxonomy. *Appl. Environ. Microbiol.* 73 5261–5267. 10.1128/AEM.00062-07 17586664 PMC1950982

[B78] Webb C. O. (2000). Exploring the phylogenetic structure of ecological communities: an example for rain forest trees. *Am. Nat.* 156 145–155. 10.1086/303378 10856198

[B79] Whitman W. B. Coleman D. C. Wiebe W. J. (1998). Prokaryotes: the unseen majority. *Proc. Natl. Acad. Sci. U.S.A.* 95 6578–6583. 10.1073/pnas.95.12.6578 9618454 PMC33863

[B80] Wilkinson T. J. Cowan A. A. Vallin H. E. Onime L. A. Oyama L. B. Cameron S. J. (2017). Characterization of the microbiome along the gastrointestinal tract of growing turkeys. *Front. Microbiol.* 8:1089. 10.3389/fmicb.2017.01089 28690591 PMC5479886

[B81] Yang L. Zhou L. Song Y. (2015). The effects of food abundance and disturbance on foraging flock patterns of the wintering *Hooded Crane* (*Grus monacha*). *Avian Res.* 6:15 10.1186/s40657-015-0024-z

[B82] Zhang B. Yang X. Guo Y. Long F. (2011). Effects of dietary lipids and *Clostridium butyricum* on the performance and the digestive tract of broiler chickens. *Arch. Anim. Nutr.* 65 329–339. 10.1080/1745039X.2011.568274 21888038

[B83] Zhang J. Lu J. (1999). Feeding ecology of two wintering geese species at poyang lake. China. *J. Freshw. Ecol.* 14 439–445. 10.1002/ece3.3566 29238566 PMC5723607

[B84] Zhao M. J. Cong P. H. Barter M. Fox A. D. Cao L. (2012). The changing abundance and distribution of greater white-fronted geese *Anser albifrons* in the yangtze river floodplain: impacts of recent hydrological changes. *Bird Conserv. Int.* 22 135–143. 10.1017/S0959270911000542

[B85] Zheng M. Zhou L. Zhao N. Xu W. (2015). Effects of variation in food resources on foraging habitat use by wintering *Hooded Cranes* (*Grus monacha*). *Avian Res.* 6:11 10.1186/s40657-015-0020-3

[B86] Zhou B. Zhou L. Chen J. Cheng Y. Xu W. (2010). Diurnal time-activity budgets of wintering *Hooded Cranes* (*Grus monacha*) in shengjin lake. China. *Waterbirds* 33 110–115. 10.1675/063.033.0114

