# Supplementary material for: Significant Differences in Bacterial and Potentially Pathogenic Communities Between Sympatric Hooded Crane and Greater White-Fronted Goose
Source: Front Microbiol. 2019 Feb 5;10:163. doi: 10.3389/fmicb.2019.00163 (PMC6370644; doi:10.3389/fmicb.2019.00163)
Supplement: Supplementary file 1 [file Table_1.DOCX]

**Supporting Information**

**Table S1:** The identified potential pathogens carried by hooded crane and greater white-fronted goose in this study.

**Table S2:** The distribution of data was analyzed by Kolmogorov-Smirnov test. Normal distribution: *P* > 0.05; Non-normal distribution: *P* < 0.05.

|  | Kolmogorov-Smirnov test | Distribution |
| --- | --- | --- |
|  | (*P* value) |  |
| Bacterial OTU richness | 0.490 | Normal |
| Bacterial phylogenitic diversity | 0.848 | Normal |
| Bacterial NTI value | 0.999 | Normal |
| Pathogenic OTU richness | 0.526 | Normal |
| Pathogenic NTI value | 0.476 | Normal |
| *Clostridium perfringens* | 0.007 | Non-normal |
| *Prevotella copri* | 0.000 | Non-normal |
| *Helicobacter pylori* | 0.000 | Non-normal |
| *Flavobacterium columnare* | 0.004 | Non-normal |
| *Plesiomonas shigelloides* | 0.001 | Non-normal |
| *Mucispirillum schaedleri* | 0.000 | Non-normal |
| *Piscirickettsia salmonis* | 0.000 | Non-normal |
| *Elizabethkingia meningoseptica* | 0.000 | Non-normal |
| *Bacillus cereus* | 0.000 | Non-normal |
| *Prevotella nigrescens* | 0.000 | Non-normal |
| *Staphylococcus aureus* | 0.000 | Non-normal |

**Table S3:** The intestinal bacterial and potentially pathogenic sequences across the samples. HC: hooded crane; GG: greater white-fronted goose.

|  | Sequences | | Pathogen/Bacteria (%) |
| --- | --- | --- | --- |
|  | Bacteria | Pathogen |  |
| HC1 | 23640 | 184 | 0.78 |
| HC2 | 16465 | 841 | 5.11 |
| HC3 | 17092 | 306 | 1.79 |
| HC4 | 12862 | 174 | 1.35 |
| HC5 | 19656 | 321 | 1.63 |
| HC6 | 22034 | 910 | 4.13 |
| HC7 | 11697 | 115 | 0.98 |
| HC8 | 15445 | 123 | 0.80 |
| HC9 | 24419 | 866 | 3.55 |
| HC10 | 14719 | 226 | 1.54 |
| HC11 | 13029 | 508 | 3.90 |
| HC12 | 13715 | 177 | 1.29 |
| HC13 | 25132 | 157 | 0.62 |
| HC14 | 11790 | 663 | 5.62 |
| HC15 | 21850 | 407 | 1.86 |
| GG1 | 14675 | 10 | 0.07 |
| GG2 | 5622 | 14 | 0.25 |
| GG3 | 8513 | 22 | 0.26 |
| GG4 | 11320 | 26 | 0.23 |
| GG5 | 12320 | 14 | 0.11 |
| GG6 | 11586 | 13 | 0.11 |
| GG7 | 13610 | 15 | 0.11 |
| GG8 | 14705 | 9 | 0.06 |
| GG9 | 11189 | 13 | 0.12 |
| GG10 | 14558 | 8 | 0.05 |
| GG11 | 14985 | 10 | 0.07 |
| GG12 | 14655 | 8 | 0.05 |
| GG13 | 6289 | 7 | 0.11 |
| GG14 | 9086 | 11 | 0.12 |
| GG15 | 16802 | 10 | 0.06 |
| Total | 443460 | 6168 | 1.39 |

**Table S4:** The differences in relative abundance of potentially pathogenic species between hooded crane and greater white-fronted goose. HC: hooded crane; GG: greater white-fronted goose. The values in brackets represent the standard deviation of the mean. Letters following brackets represent significant differences from Mann-Whitney-Wilcoxon test (*P* < 0.05).

**
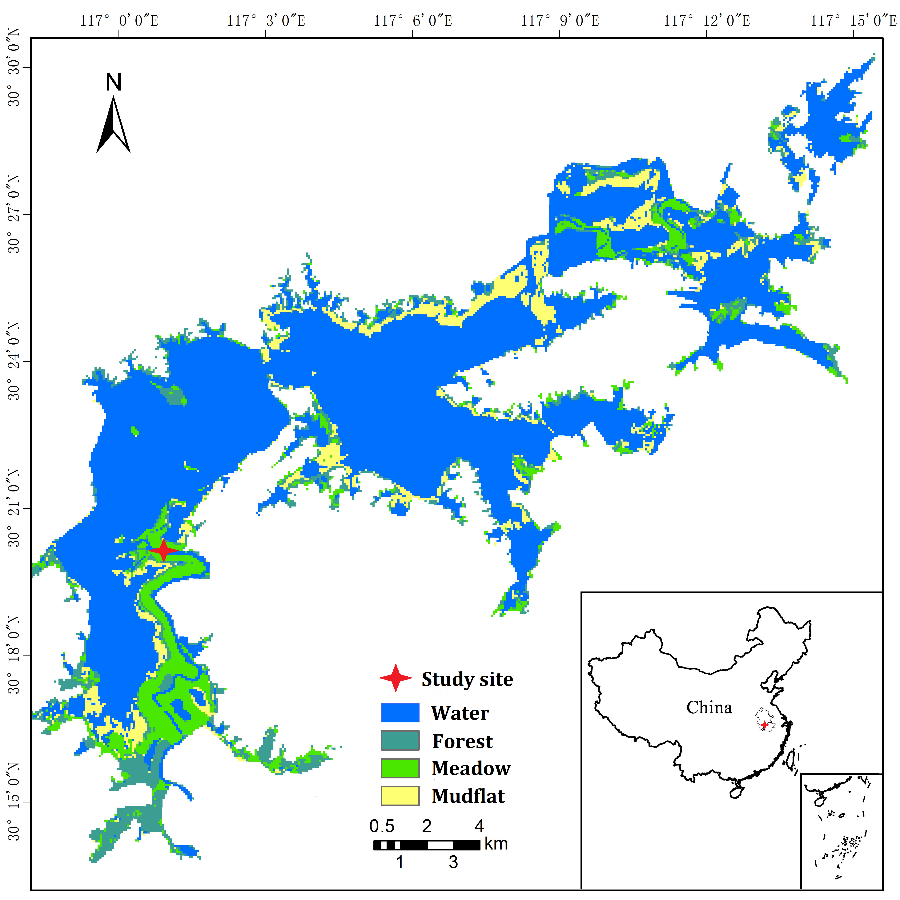
Fig. S1:** A schematic of study area.

**
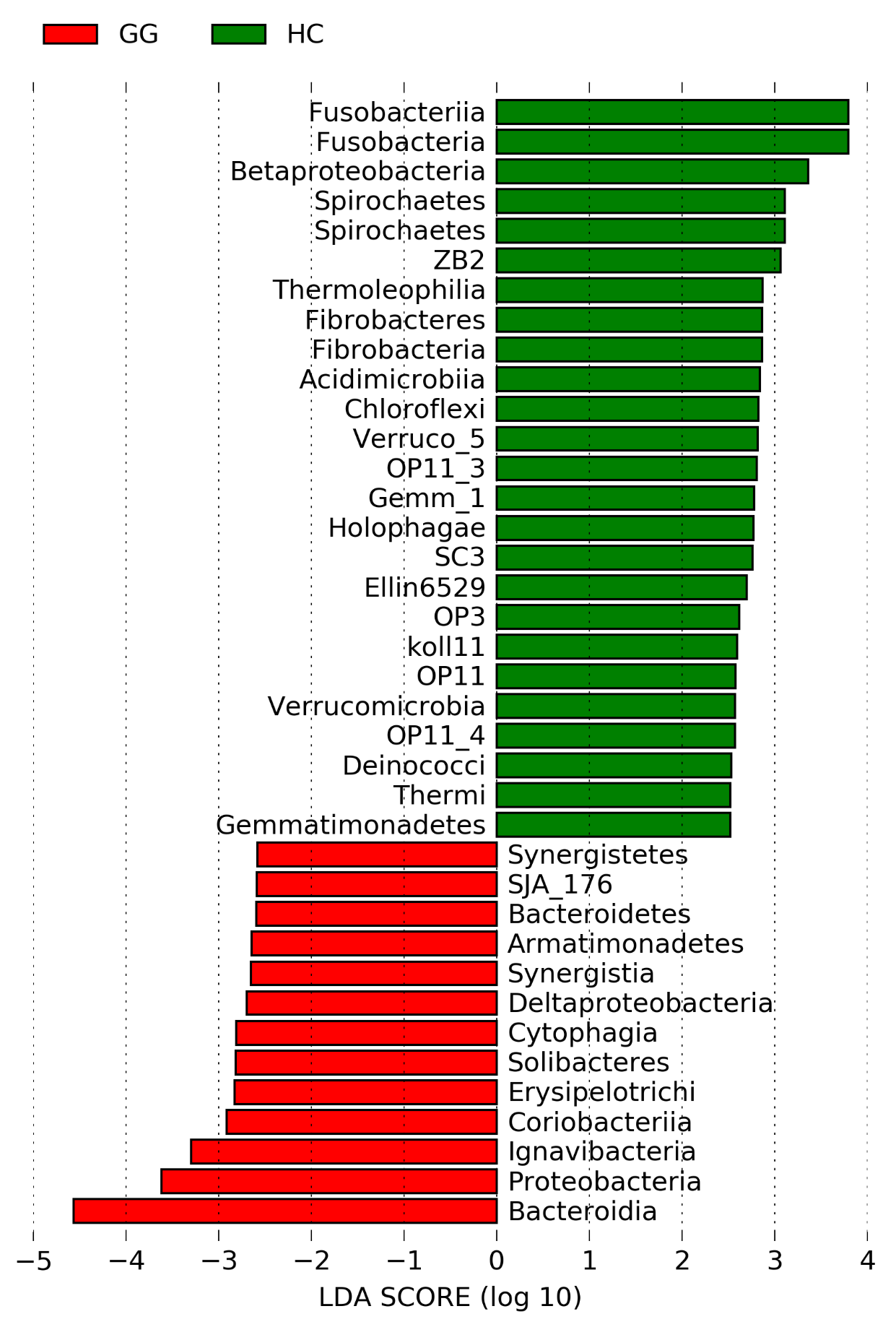
Fig. S2:** Identified phylotype biomarkers ranked by effect size in HC and GG. The phylotype biomarkers were identified as being significantly abundant when samples from HC and GG were compared and the alpha value was <0.05. HC: hooded crane; GG: greater white-fronted goose.

**
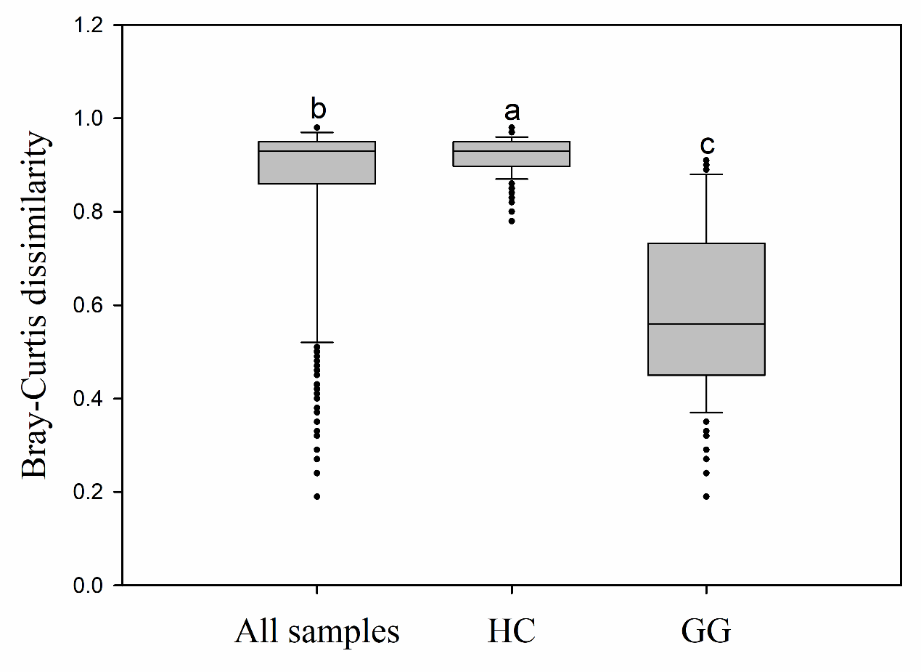
Fig. S3:** The comparison of bacterial Bray-Curtis dissimilarity among different treatments. HC: hooded crane; GG: greater white-fronted goose.
